# Impact of subthreshold depression on health-related quality of life in patients with Parkinson’s disease based on cognitive status

**DOI:** 10.1186/s12955-021-01753-5

**Published:** 2021-03-25

**Authors:** Aline Schönenberg, Hannah M. Zipprich, Ulrike Teschner, Julian Grosskreutz, Otto W. Witte, Tino Prell

**Affiliations:** 1 Department of Neurology, Jena University Hospital, Am Klinikum 1, 07747 Jena, Germany; 2 Center for Healthy Ageing, Jena University Hospital, Jena, Germany

**Keywords:** Subthreshold depression, Dementia, Quality of life, Parkinson’s disease, Cognitive impairment, Non-motor symptoms

## Abstract

**Background:**

In patients with Parkinson’s disease (PD), depression has a strong impact on quality of life (QoL). However, little is known about the influence of subthreshold depression (STD) on QoL in PD patients.

**Methods:**

A total of 230 hospitalized PD patients with normal and impaired cognitive status were included in this observational study. We collected the following data for analysis: Beck Depression Inventory level, Montreal Cognitive Assessment (MOCA) score, non-motor symptoms questionnaire score, PD questionnaire-39 (PDQ-39) score, Hoehn–Yahr stage, and Movement Disorder Society-sponsored revision of the unified PD rating scale III (MDS-UPDRS III) score. To study the impact of STD on the PDQ-39 summary index (SI) and its domains, we used multivariate analysis of variance and multivariate analysis of covariance.

**Results:**

In this cohort, 80 (34.8%) patients had STD [44 (32.3%) with high MOCA score (> 21) and 36 (38.3%) with low MOCA score (< 21)]. In PDQ-39 SI, there was a significant effect on depression level. In patients with higher MOCA score, STD was associated with worse PDQ-39 domains emotional well-being and cognition, whereas in patients with lower MOCA score, STD had no significant effect on PDQ-39 SI or its subdomains.

**Conclusion:**

In PD patients, QoL is significantly affected by STD, and thus, more attention in medical care should be focused on treating STD. However, the impact is only observable in PD patients with normal cognitive function. STD patients show more reduced QoL than non-depressed patients, indicating that STD should be treated as a transition zone between normal mood and depression.

**Supplementary Information:**

The online version contains supplementary material available at 10.1186/s12955-021-01753-5.

## Background

Besides motor symptoms, a plethora of non-motor symptoms (NMS) characterizes Parkinson’s disease (PD). Among the NMS, depression and cognitive decline are quite common [1]. One of the greatest influences on the quality of life (QoL) of PD patients is depression, accounting for up to 60% of the variability in QoL [2–5]. Depressive symptoms manifest along a continuum. In PD patients, the importance of depression was often investigated; however, little is known about subthreshold (also subsyndromal) depression (STD). Although STD in PD patients has high prevalence rates of 21.0%–28.8% [6–8], there is no generally accepted definition of STD. It is commonly described as depressive symptoms without fulfilling the diagnostic criteria for either mild or severe depression or dysthymia [9, 10]. Generally, STD patients show more symptoms and impairment than non-depressed patients, and these STD-related impairments are clinically relevant [9]. Compared with non-depressed patients, STD patients show increased individual complaints, resulting in poorer well-being [10]. Moreover, STD can precede major depression [11].

So far, little is known about the association between STD and QoL. One study found a strong linear decline of QoL (assessed with the PD questionnaire-39, PDQ-39) with increasing depression level (Beck Depression Inventory, BDI II). In comparison with non-depressed patients, STD patients showed poorer overall QoL [PDQ-39 summary index (SI)] and poorer PDQ-39 subdomains mobility, emotional well-being, stigmatization, cognition, and communication [7]. However, the cohort of outpatients with unimpaired cognitive function does not reflect the entire spectrum of PD patients [7]. As cognitive decline and PD dementia are common in PD and are relevant predictors of QoL [12–14], an analysis of the association between depression and QoL in PD patients with different degrees of cognitive decline is necessary.

Therefore, this study explored the association between STD and QoL in PD patients with and without cognitive impairment.

## Methods

### Participants and assessments

This study was conducted under the Declaration of Helsinki and was approved by the Ethics Committee of the Jena University Hospital. Written informed consent was obtained from all enrolled patients. According to the Movement Disorder Society (MDS) diagnostic criteria, PD patients were consecutively recruited between January 2018 and January 2020 from the ward of the Department of Neurology, Jena University Hospital, Germany. Patients were excluded if they have delirium or severe dementia with an inability to understand and complete a questionnaire. All tests were conducted during on-phase medication. We screened 250 patients, from which 20 patients were excluded because of severe delirium (13 patients) or missing data (7 patients). Finally, a total of 230 patients were included in the analysis.

The data was collected by trained research staff. Using the Montreal Cognitive Assessment (MOCA), we assessed the cognitive status after a short introduction to the aims and methods of the study [15]. A valid impression of each patient’s ability to understand and complete a questionnaire was achieved with face-to-face testing. Thus, patients with a MOCA score below the common threshold of 21 points for PD dementia [16] were included if they could understand and answer the questionnaires coherently. Then, we collected the following demographic and clinical data: age, gender, marital status (single, divorced, widowed, or married), level of education [high (German Abitur or University), medium (German Realschule or General Certificate of Secondary Education), low (German Hauptschule), or no school], the total daily number of medication administered in any pharmaceutical form, Beck Depression Inventory-II [17], MDS-sponsored revision of the unified PD rating scale III (MDS-UPDRS III) [18], revised NMS questionnaire (NMS-Q) [19], Hoehn–Yahr (HY) stage [20], and health-related QoL of the PDQ-39. The PDQ-39 is a questionnaire devised specifically for the assessment of QoL in PD patients, which consists of an SI and eight subdomains concerning mobility, activities of daily living, emotional well-being, stigmatization, social support, cognition, communication, and bodily discomfort [21].

### Statistics

Statistical analyses were performed using SPSS version 25.0 (IBM, New York, NY, USA), with a *p* value < 0.05 indicating statistical significance. Values are given as mean and standard deviation or median and interquartile range, and categorical variables are presented as numbers or percentages.

The cohort was categorized according to the depression level [non-depressed (BDI II < 9), STD (BDI II 9–15), and depressed (BDI II > 15) [7] and cognitive status based on the MOCA score [normal cognition (30–26 points), mild cognitive impairment (21–25 points), and dementia (< 21 points)] [16].

In the first step, using descriptive statistics, we analyzed the cohort, and by using the Shapiro–Wilk test, we determined the normal distribution. Group comparisons were performed with the Kruskal–Wallis test with Bonferroni correction for metric values and chi-square or exact test for categorical variables.

To study the association between several clinical parameters (age, gender, education level, HY stage, MDS-UPDRS III, NMS-Q, MOCA, BDI II) and the PDQ-39 SI (backward selection), we performed a multivariable linear regression. The significance levels for variables entering the linear regression model and for removing from the model were set at 0.05 and 0.1, respectively. Before regression analyses, we excluded autocorrelation (Durbin–Watson) and multicollinearity (variance inflation factor and tolerance). Power analysis showed that the overall model with eight predictors is significant with a coefficient of determination of *R*^2^ = 0.5, a statistical power of 0.95, a significance level of α = 0.05, and a sample size of *n* = 32.

To study the effect of depression level based on the BDI II score on the PDQ-39 domains, we used multivariate analysis of variance (MANOVA). There were low correlations between dependent variables (*r* < 0.90), indicating that multicollinearity was not a confounding factor in the analysis. As assessed by the Mahalanobis distance and removed from the following analysis, we found two multivariate outliers. As assessed by Levene’s test, we found homogeneity of the error variances for PDQ-39 domains (*p* > 0.05), but not for social support. As assessed by the Box’s test, we found homogeneity of covariances. Thus, to adjust these findings for other clinical variables, we used multivariate analysis of covariance (MANCOVA).

## Results

The cohort included 91 (39.6%) female and 139 (60.4%) male PD patients with a mean age of 70.5 years [standard deviation (SD) = 8.5]. Since the first PD motor symptoms, the mean disease duration was 7.6 years (SD = 5.5); 10 (4.3%) patients were in HY stage I, 37 (16.1%) patients in HY stage II, 141 (61.3%) patients in HY stage III, 37 (16.1%) patients in stage IV, and 5 (2.2%) patients in stage V. Given that we had no preliminary data about the impact of cognition on the association between QoL and STD, we analyzed the patients with MOCA scores < 21 points (*n* = 94, 41.2%) and patients with MOCA ≥ 21 points (*n* = 136, 59.1%) separately.

### Association between PDQ-39 and depression in patients with MOCA scores ≥ 21

In PD patients with MOCA scores ≥ 21 points, 56 (41.2%) patients were classified as non-depressed, 44 (32.4%) patients had STD, and 36 (26.5%) patients were depressed. These three groups did not differ in age, disease duration, MDS-UPDRS III, and MOCA. However, in comparison with non-depressed patients, STD patients had higher NMS-Q scores and higher HY stages (Table 1).

**Table 1:** Clinical and sociodemographic characteristics of patients with MOCA score ≥ 21 based on depression level

	Nondepressed		Subthreshold depression		Depression		*p*
	*n*	%	*n*	%	*n*	%	
*Sex*							
Female	18	16.7	14	31.8	17	47.2	0.21
Male	38	67.9	30	68.2	19	52.8	
*Education level*							
Low	9	17.0	5	12.2	3	9.7	0.40
Middle	14	26.4	16	39.0	15	48.4	
High	30	56.6	20	48.8	13	41.9	

	Nondepressed		Subthreshold depression		Depression		*p*
	M	SD	M	SD	M	SD	
Age (years)	68.8	8.3	68.1	7.9	67.7	8.0	0.697
Disease duration (years)	7.9	5.1	6.7	5.2	7.1	5.7	0.463
Hoehn and Yahr stage	3 ^*a*^	1	3 ^*b*^	0	3 ^*c*^	0	0.009
MDS-UPDRS III	22.6	12.3	25.9	12.2	28.4	15.6	0.176
NMS-Quest	7.4^*a*^	4.1	9.7^*b*^	3.8	14.1^*c*^	4.9	< 0.001
BDI II II	5.2^*a*^	2.3	11.5^*b*^	2.0	23.6^*c*^	8.3	< 0.001
MOCA	24.8	2.5	24.2	2.6	23.9	2.3	0.183

Data are presented as mean and SD and for Hoehn and Yahr as median and IQR. Data were analyzed using the Kruskal–Wallis test with Bonferroni-correction. Post-hoc tests for NMS-Q *ab* *p* = 0.043, *bc* *p* = 0.004, *ac* *p* < 0.001; for BDI II *ab* *p* < 0.001, *bc* *p* < 0.001, *ac* *p* < 0.001; for Hoehn and Yahr stage *ab* *p* = 0.03, *bc* *p* = 0.4, *ac* *p* = 0.004.

**Fig. 1:**
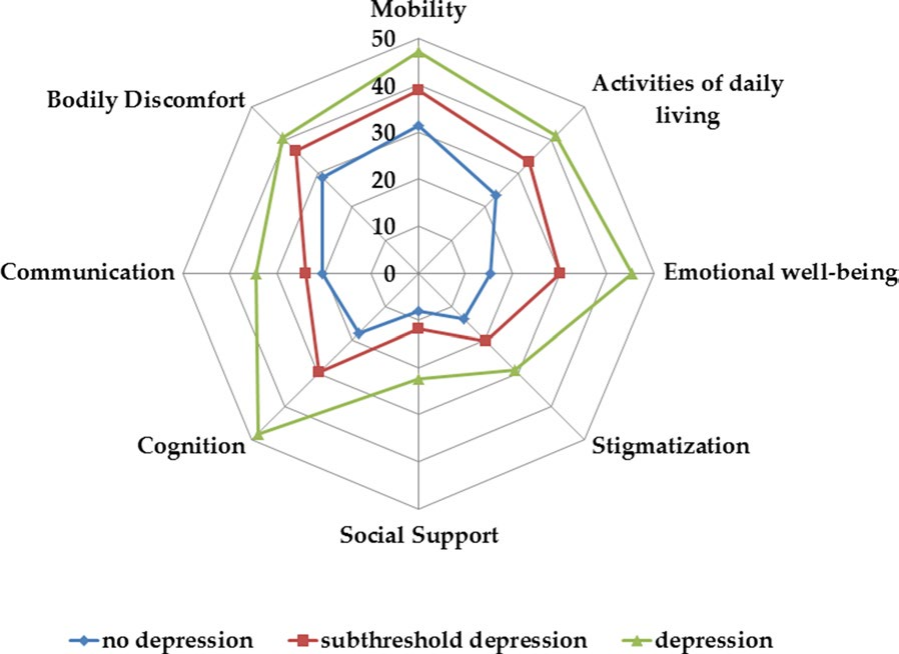
Polar plot for the different PDQ-39 domains for patients without depression, with subthreshold depression and with depression (in the subgroup of patients with normal MOCA)

The PDQ-39 SI differed significantly between the non-depressed patients (mean = 19.9, 95% confidence interval (CI) [16.3, 23.4]) and the STD patients (mean = 28.4, 95%CI [23.7, 33.1]) as well as between the STD and depression group (mean = 37.8, 95%CI [31.1, 44.5]). Figure 1 shows the different PDQ-39 domains in patients with depression, with STD, and without depression (detailed values are shown in Additional file 1: Table S1). STD patients scored better in the PDQ-39 domain cognition (*p* = 0.042) than depressed patients. On the other hand, STD patients showed poorer QoL in the domain emotional well-being (*p* = 0.003) than non-depressed patients.

The PDQ-39 SI was predicted by the BDI II (standardized β = 0.246, *p* = 0.004), NMS-Q (standardized β = 0.466, *p* < 0.001), and MDS-UPDRS III (standardized β = 0.162, *p* = 0.024) (*F*(3, 110) = 33.4, *p* < 0.001, adjusted *R*^2^ = 0.46) (Additional file 1: Table S2a); 27% of the PDQ-39 SI variance is attributed to the BDI II alone. As the BDI II-II and the PDQ-39 subdomain emotional well-being measure similar constructs and correlated significantly (r = 0.547, *p* < 0.001), we provide an additional analysis (F(4, 54) = 17.0, *p* < 0.001) with the exclusion of the PDQ-39 emotional well-being subdomain in the supplementary materials (Additional file 1: Table S2b). With the exclusion of the emotional well-being subdomain from the PDQ-39, the BDI II was no longer significant in the analysis. However, in addition to NMS-Q and MDS-UPDRS III, the MOCA became a significant predictor (standardized ß = − 0.228, *p* = 0.02).

On the basis of the BDI II score, to analyze the impact of depression level (non-depressed, STD, and depressed) on the eight PDQ-39 domains, we performed MANOVAs. A one-way MANOVA found statistically significant differences between the depression levels on the combined dependent variables [*F*(16, 234) = 4.34, *p* < 0.001, partial η^2^ = 0.23, and Wilk’s λ = 0.59]. For every dependent variable, we conducted post-hoc univariate ANOVAs. Results showed a statistically significant difference between the depression levels for activity of daily living [*F*(2, 124) = 5.3, *p* = 0.006, partial η^2^ = 0.08], emotional well-being [*F*(2, 124) = 26.6, *p* < 0.001, partial η^2^ = 0.30], stigmatization [*F*(2, 124) = 3.1, *p* = 0.050, partial η^2^ = 0.047], social support [*F*(2, 124) = 3.6, *p* = 0.03, partial η^2^ = 0.055], cognition [*F*(2, 124) = 22.9, *p* < 0.001, partial η^2^ = 0.27], and communication [*F*(2, 124) = 3.4, *p* = 0.036, partial η^2^ = 0.05], but not for mobility and bodily discomfort. Tukey’s honestly significant difference (HSD) post-hoc analysis on PDQ-39 domains revealed a significant difference between non-depressed patients and STD patients for emotional well-being (*M*_Diff_ =  − 52.7, 95%CI [− 80.6, − 24.7]) and cognition (*M*_Diff_ =  − 38.3, 95% CI [− 66.5, − 10.1]), but not for other PDQ-39 domains (Additional file 1: Table S3). Moreover, to examine whether the previously identified significant predictors of the PDQ-39, MDS-UPDRS III and NMS-Q, could account for these findings, we conducted MANCOVAs, in which we found that depression level (Wilks’s λ = 0.71, *p* = 0.003, partial η^2^ = 0.155), MDS-UPDRS III (Wilks’s λ = 0.84, *p* = 0.02, partial η^2^ = 0.16), and NMS-Q (Wilks’s λ = 0.54, *p* < 0.001, partial η^2^ = 0.46) were significant in the model. After the addition of MDS-UPDRS III and NMS-Q, a significant difference between the three depression levels for emotional well-being (*p* = 0.001, partial η^2^ = 0.12) and cognition (*p* = 0.01, partial η^2^ = 0.08) still remained.

### Association between PDQ-39 and depression in patients with MOCA scores < 21

**Fig. 2:**
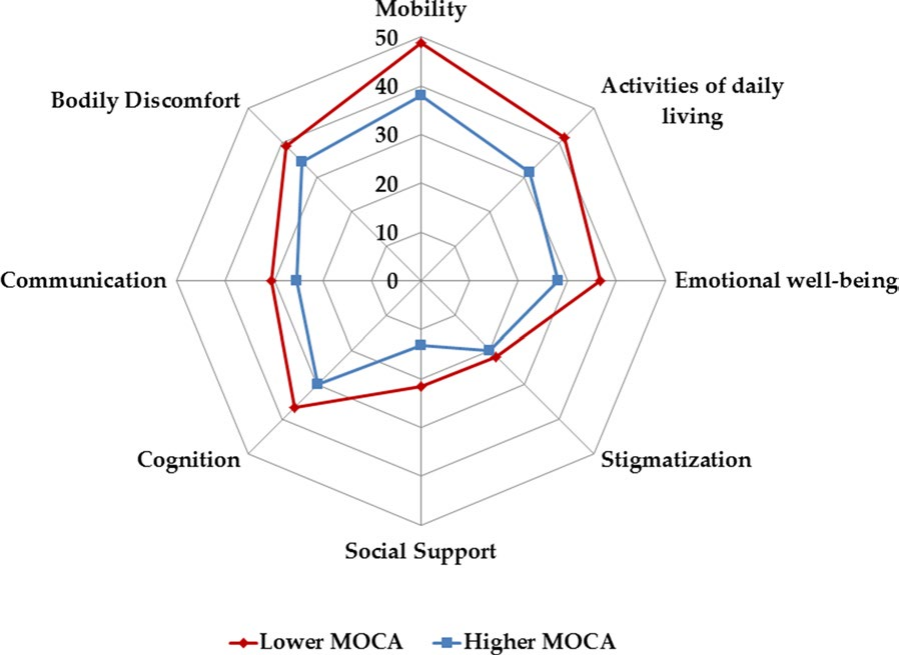
Polar plot for the different PDQ-39 domains depending on MOCA scores

In PD patients with MOCA scores < 21 points, the same analyses were performed. In this subgroup, 21 (22.3%) patients were classified as non-depressed, 36 (38.3%) patients had STD, and 37 (39.4%) patients were depressed (for a detailed description of this cohort, see Additional file 1: Table S4). When compared with patients with higher MOCA score (Additional file 1: Table S5), PD patients with lower MOCA were characterized by higher age (*p* < 0.001), higher MDS-UPDRS III (*p* < 0.001), higher NMS-Q (*p* = 0.01), higher BDI II (*p* = 0.007), and higher HY stage (chi-square *p* = 0.02). The duration of the disease did not differ between both groups (Additional file 1: Table S4). The PDQ-39 domains mobility (*p* = 0.01), ADL (*p* = 0.02), social support (*p* = 0.003), cognition (*p* = 0.014), and communication (*p* = 0.03) differed significantly between the higher and lower MOCA groups (Fig. 2).

STD patients scored better in the PDQ-39 domains mobility (*p* = 0.02), emotional well-being (*p* < 0.001), and communication (*p* = 0.003) than depressed patients. The eight PDQ-39 domains did not significantly differ between non-depressed patients and STD patients.

The PDQ-39 SI was predicted by the BDI II (standardized β = 0.247, *p* = 0.024), gender (standardized β =  − 2.42, *p* = 0.022), MDS-UPDRS III (standardized β = 0.206, *p* = 0.058), NMS-Q (standardized β = 0.39, *p* = 0.001), and MOCA (standardized β = 0.228, *p* = 0.034) *F*(5, 66) = 7.5, *p* < 0.001, adjusted *R*^2^ = 0.32) (Additional file 1: Table S6a); 13% of the PDQ-39 SI variance is attributed to the BDI II alone. As within the cohort of higher MOCA scores, an additional analysis (F(3, 34) = 10.5, *p* < 0.001) with the exclusion of the PDQ-39 emotional well-being domain is provided in the supplementary materials (Additional file 1: Table S6b). With the exclusion of the emotional well-being subdomain, both the BDI II (standardized ß = 0.324, *p* = 0.019) and the NMS-Q (standardized ß = 0.379, *p* = 0.015)) remained significant.

A one-way MANOVA showed statistically significant differences between the depression levels on the combined dependent variables [*F*(16, 168) = 2.66, *p* = 0.001, partial η^2^ = 0.20, Wilk’s λ = 0.63]. Post-hoc univariate ANOVAs showed a statistically significant difference between the depression levels for mobility, ADL, emotional well-being, social support, and communication (Additional file 1: Table S7)*.* Tukey’s HSD post-hoc analysis on PDQ-39 domains revealed no significant difference between non-depressed patients and STD patients for any PDQ-39 domains. After the addition of MDS-UPDRS (Wilks’s λ = 0.76, *p* = 0.007, partial η^2^ = 0.24) and NMSQ (Wilks’s λ = 0.75, *p* = 0.004, partial η^2^ = 0.25) to the model, the MANCOVA still presented a significant effect of the depression levels on the PDQ-39 domains (Wilks’s λ = 0.62, *p* = 0.002, partial η^2^ = 0.21) with significant differences for emotional well-being (*p* < 0.001, partial η^2^ = 0.17), social support (*p* = 0.002, partial η^2^ = 0.15), and communication (*p* = 0.01, partial η^2^ = 0.10). The difference between non-depressed and depressed patients has driven this effect.

**Fig. 3:**
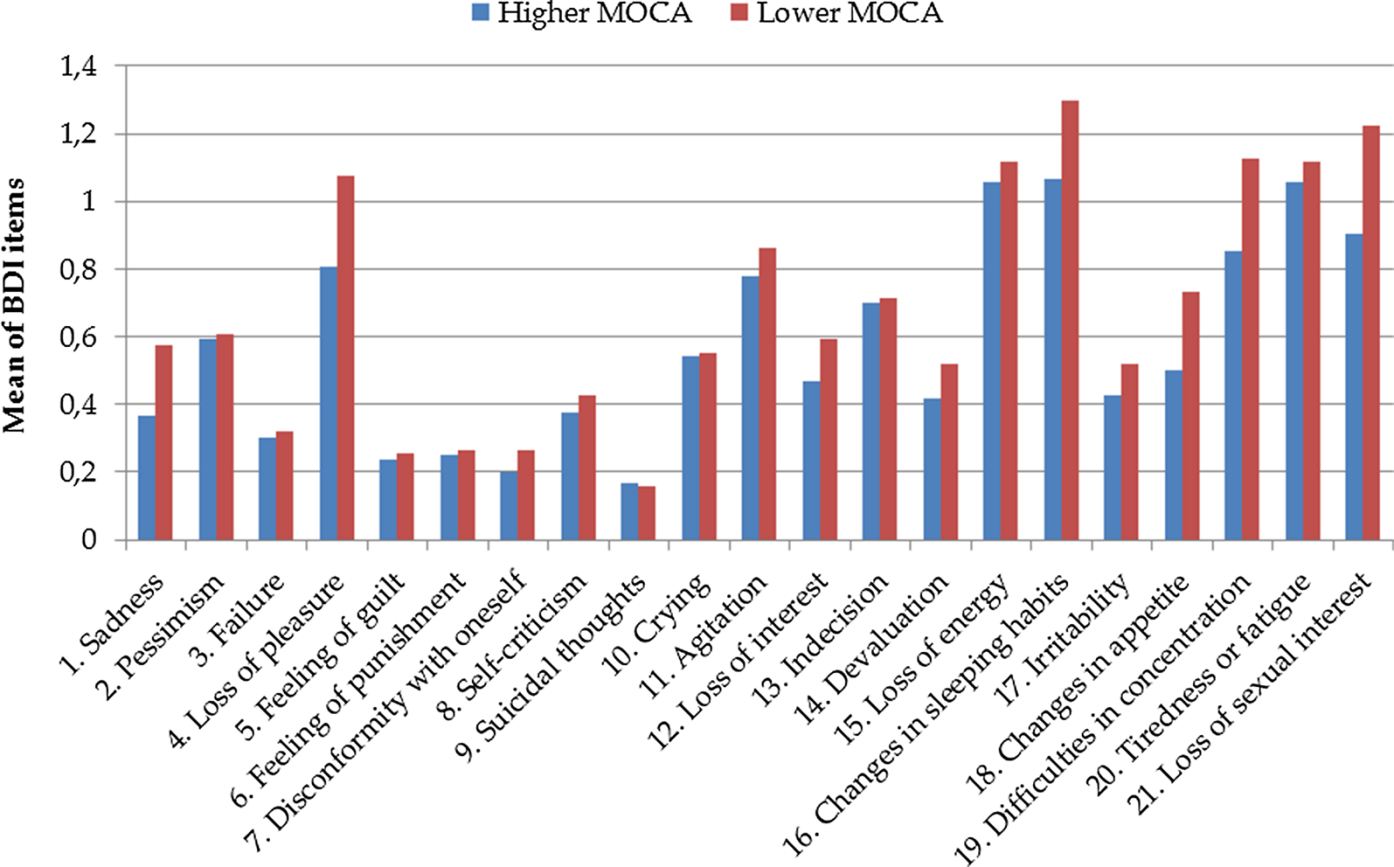
Responses to the BDI II items in PD patients with higher and lower MOCA scores

Finally, Fig. 3 shows the responses to the BDI II items in PD patients with higher and lower MOCA scores. Using a lower threshold of statistical significance (*p* < 0.001), we found that the mean of each 21 BDI II items did not differentiate the two groups, indicating that both groups show comparable responsiveness to the BDI II.

## Discussion

In the present study, 80 (34.8%) PD patients had STD with higher prevalence in the group with a lower MOCA score. Because of different definitions of STD, age, disease duration, and cognitive status, this rate is slightly higher than in other studies, which reported prevalence rates between 21.0 and 28.8% [6–8]. Our results indicate that for patients with normal cognitive function, STD impacts overall QoL, as measured by the PDQ-39 SI, and distinct PDQ-39 domains, which is partly supported by Reiff et al. [7] in outpatients. However, only group comparisons were used to detect differences in PDQ-39 domains in their study. This missing correction for other cofactors could explain why they also observed changes in mobility, stigma, and communication when comparing non-depressed patients with STD patients. In our cohort, STD was only associated with emotional well-being and cognition after correction for motor function and non-motor burden. Generally, the QoL of STD patients was situated between the QoL of non-depressed and depressed PD patients, indicating that STD should be regarded as a transition zone between a non-depressed and depressed state.

In PD patients with lower MOCA scores, there was an effect of depression level on PDQ-39 SI and certain PDQ-39 domains. However, there was no significant effect between non-depressed patients and STD patients, indicating that these effects were driven by depressed patients only. One can conclude that STD impacts the PDQ-39 domain emotional well-being and cognition in patients with normal cognitive function but not in patients with cognitive deficits. In patients with lower MOCA scores, QoL was only influenced by severe depressive symptoms. Thus, STD seems to modulate QoL differently depending on cognitive function. These findings, however, need methodological considerations.

First of all, it seems counterintuitive that the effect of cognition on QoL is significant for patients with higher MOCA scores, but not for patients with lower MOCA scores. However, the PDQ-39 subdomain cognition is more related to mood and depression rather than other neuropsychological measurements. Thus, the PDQ-39 cognition domain is not a measure of cognitive function and not necessarily influenced by the current cognitive ability. The PDQ-39 domain emotional well-being, which is influenced by STD in the current study, is primarily associated with depression and anxiety, which is in line with the concept of QoL being strongly related to depression [22]. As expected and demonstrated by previous studies, there is an overlap and correlation between depression and emotional well-being [23, 24]. Therefore, we have analyzed factors relating to the PDQ-39 sum score both with and without the emotional well-being subdomain. Our results show that for patients with higher MOCA scores, the impact of the BDI II on the PDQ-39 vanishes after removing the emotional well-being domain. This indicates that for these patients, depression was mainly related to emotional well-being. Interestingly, the MOCA only emerged as a significant indicator after exclusion of the emotional well-being subdomain, suggesting that, in line with our other results, the effect of cognition on QoL is strongly related to emotional well-being.

After exclusion of the emotional well-being subdomain from the PDQ-39 in patients with lower MOCA scores, the significant influence of the MOCA on the PDQ-39 vanished, whereas the BDI II-II and NMS-Q remained significant predictors of the PDQ-39. Again, these results serve to further highlight the strong relation between cognition and emotional well-being. Furthermore, the effect of the BDI II-II suggests that, unlike for patients with higher MOCA, depression was primarily related to physical problems in this cohort, which is in line with the higher age, higher HY stage and higher MDS-UPDRS III scores in this subgroup [25].

Our results indicate that cognitive and somatic factors of the BDI II-II [26] are differentially influenced in our two MOCA groups. As both depression and QoL are highly individual and multi-faceted constructs [27], it is necessary to keep in mind that those constructs may overlap and mutually influence each other [24]. Therefore, it is not possible to fully separate questionnaires that measure overlapping symptoms, such as the BDI II-II and the PDQ-39. However, we believe that our results hold since we used highly valid questionnaires [17, 28] and controlled for possible overlaps in our analysis.

Secondly, unlike previous studies, this study did not strictly exclude patients with lower MOCA scores. This approach has been chosen because it is problematic to exclude patients with cognitive dysfunction who are so common in PD [13]. To our understanding and impression from face-to-face interaction, all patients could complete and understand the questionnaires. Whether cognitively impaired patients can reliably fill out a questionnaire the question has been discussed in many ways before and results indicate that assessment of QoL via self-report measures is possible and valid independent of cognitive impairment or age [29]. This is in line with the result that, on average, patients with normal and lower MOCA scores responded comparably to the BDI II items in the current study (Fig. 3). In both groups, BDI II was the strongest predictor of QoL, underlining the prominent effect of depression and NMS on QoL in PD [2, 4, 5].

Thirdly, the comparability of previous studies is reduced by the missing clear definition of STD, as it was operationalized and measured differently in each study [6–8]. Given the impact of STD on QoL, an agreement on the standardized definition of STD is needed to make research comparable. Although the BDI II is recommended in detecting depression in PD and in differentiating between actual PD symptoms and symptoms of depression independent of PD [17, 30, 31], it poses the limitation as it has a focus on somatic symptoms. The separation of depressive symptoms and other PD-related NMS is crucial to understanding how different NMS contribute to poor QoL. It can be hard to distinguish symptoms like fatigue, difficulty in sleeping and concentrating, or loss of appetite in relation to depression, PD, or both [8]. In agreement with Reiff et al. [7], we argue that the burden and the effect of these symptoms on QoL remain the same, whether attributed to PD or depression. Therefore, treatment of these symptoms should not be hindered by a lack of clarity on their etiology, especially because evidence suggests that depression does not differ between PD patients and other depressed patients [32] and that depression and PD symptoms can be separated [30]. Furthermore, indicators of the disease itself, such as severity, duration, and HY stage, did not have a significant influence in our study, underlining that depression is an independent predictor of QoL in PD.

Finally, even though research repeatedly shows that depression strongly impacts QoL of PD patients [2, 4, 33], it is important to shed further light on the influence of depression on patients' well-being due to an under-recognition of depressive symptoms in PD by neurologists [34]. It is crucial to heighten awareness for those symptoms at a subthreshold level because STD reduces QoL and is associated with increased risk for developing major depression [11]. Depressive symptoms influence not only QoL but also disability and disease severity [33, 35, 36] and thus the progression of PD itself, highlighting the importance of identifying depressive symptoms early, at a subthreshold level, to stabilize the patients' QoL and to obviate the possible aggravation of depression and PD itself. Therefore, further research to investigate treatment and therapy for STD is needed.

This study is not free of limitations. The cross-sectional design does not allow any conclusion on causality. Its generalizability is reduced by selecting people from a specialized movement disorder ward in a university hospital. Although the results fit the study by Reiff et al. (7), the results cannot generalize community-dwelling PD patients.

## Supplementary Information

**Additional file 1.** Detailed statistical analyses.

## Data Availability

The datasets used and/or analysed during the current study are available from the corresponding author on reasonable request.
