# Supplementary material for: Impact of subthreshold depression on health-related quality of life in patients with Parkinson’s disease based on cognitive status
Source: Health Qual Life Outcomes. 2021 Mar 25;19:107. doi: 10.1186/s12955-021-01753-5 (PMC7993461; doi:10.1186/s12955-021-01753-5)
Supplement: Supplementary file 1 — Additional file 1. Detailed statistical analyses. [file 12955_2021_1753_MOESM1_ESM.docx]

**Additional file 1**

**Table S1.** PDQ-39 domains in patients with normal and low Montreal cognitive assessment (MOCA) scores.

|  | **Non-depressed** | | | | **Subthreshold Depression** | | | | **Depression** | | | |
| --- | --- | --- | --- | --- | --- | --- | --- | --- | --- | --- | --- | --- |
| **Patients with MOCA <21** | | | | | | | | | | | | |
|  | M | SD | 95%CI | | M | SD | 95%CI | | M | SD | 95%CI | |
| Mobility | 39.9 | 27.1 | 27.6 | 52.2 | 44.0 | 26.3 | 35.1 | 52.9 | 58.4 | 24.5 | 50.3 | 66.6 |
| Activities of daily living | 33.1 | 23.4 | 22.5 | 43.8 | 37.4 | 23.0 | 29.6 | 45.2 | 50.2 | 26.3 | 41.4 | 58.9 |
| Emotional well-being | 27.2 | 19.4 | 18.4 | 36.1 | 30.4 | 19.3 | 23.9 | 36.9 | 48.5 | 17.6 | 42.7 | 54.4 |
| Stigmatization | 16.0 | 14.8 | 9.3 | 22.8 | 23.4 | 22.8 | 15.7 | 31.1 | 23.7 | 18.8 | 17.4 | 29.9 |
| Social Support | 10.7 | 18.5 | 2.3 | 19.1 | 17.1 | 19.5 | 10.5 | 23.7 | 32.2 | 22.9 | 24.6 | 39.9 |
| Cognition | 29.8 | 18.6 | 21.3 | 38.3 | 34.0 | 18.9 | 27.6 | 40.4 | 42.7 | 21.3 | 35.6 | 49.8 |
| Communication | 28.2 | 19.5 | 19.3 | 37.1 | 22.1 | 20.1 | 15.3 | 28.9 | 40.3 | 21.4 | 33.2 | 47.5 |
| Bodily Discomfort | 33.8 | 24.2 | 22.7 | 44.8 | 38.2 | 22.3 | 30.7 | 45.8 | 42.4 | 22.0 | 35.0 | 49.7 |
| PDQ-39 Summary index | 27.3 | 15.3 | 20.3 | 34.3 | 30.9 | 14.2 | 26.1 | 35.7 | 42.3 | 14.6 | 37.5 | 47.2 |
| **Patients with MOCA ≥21** | | | | | | | | | | | | |
|  | M | SD | 95%CI | | M | SD | 95%CI | | M | SD | 95%CI | |
| Mobility | 31.3 | 27.3 | 24.0 | 38.7 | 38.9 | 25.5 | 31.0 | 46.9 | 47.2 | 28.2 | 37.7 | 56.8 |
| Activities of daily living | 23.4 | 21.5 | 17.7 | 29.2 | 33.3 | 24.0 | 26.0 | 40.6 | 41.3 | 28.7 | 31.6 | 51.1 |
| Emotional well-being | 15.3 | 16.7 | 10.8 | 19.8 | 30.2 | 18.7 | 24.5 | 35.9 | 45.4 | 23.2 | 37.6 | 53.3 |
| Stigmatization | 13.7 | 17.4 | 9.0 | 18.4 | 20.4 | 21.1 | 14.0 | 26.8 | 29.0 | 26.1 | 20.2 | 37.9 |
| Social Support | 8.1 | 13.1 | 4.5 | 11.7 | 11.7 | 16.2 | 6.8 | 16.6 | 22.3 | 25.5 | 13.6 | 31.1 |
| Cognition | 17.9 | 15.9 | 13.6 | 22.3 | 29.7 | 21.0 | 23.2 | 36.2 | 48.2 | 21.7 | 40.7 | 55.6 |
| Communication | 20.5 | 21.8 | 14.5 | 26.4 | 23.9 | 22.4 | 16.9 | 30.9 | 34.5 | 25.9 | 25.6 | 43.4 |
| Bodily Discomfort | 28.8 | 22.9 | 22.6 | 35.1 | 36.7 | 26.7 | 28.4 | 45.0 | 40.7 | 21.7 | 33.2 | 48.1 |
| PDQ-39 Summary index | 19.9 | 12.8 | 16.3 | 23.4 | 28.4 | 15.0 | 23.7 | 33.1 | 37.8 | 19.2 | 31.1 | 44.5 |

**Table S2a.** Linear regression (backward selection): Association with PDQ-39 Summary Index in patients with normal Montreal cognitive assessment (MOCA ≥ 21)**.**

|  | | **Unstandard. coefficient** | **Standard error** | **Standard. coefficient β** | **t** | ***p*** | **Lower 95%CI** | **Upper 95%CI** | **Corrected R^2^** |
| --- | --- | --- | --- | --- | --- | --- | --- | --- | --- |
|  | constant | .963 | 3.129 |  | .308 | .759 | -5.238 | 7.163 | 0.463 |
|  | BDI II | .476 | .162 | .246 | 2.933 | .004 | .154 | .797 |  |
|  | NMS-Q | 1.568 | .288 | .466 | 5.448 | .000 | .997 | 2.138 |  |
|  | MDS-UPDRS-III | .206 | .090 | .162 | 2.287 | .024 | .027 | .384 |  |

Entered independent variables: Beck Depression Inventory II (BDI II), Montreal cognitive assessment (MOCA), non-motor symptoms questionnaire (NMS-Q), MDS-sponsored revision of the unified PD rating scale III (MDS-UPDRS-III), Hoehn & Yahr stage (HY), disease duration, sex, age, education level.

**Table S2b.** Linear regression (backward selection): Association with PDQ-39 Summary Index excluding emotional well-being subdomain in patients with normal Montreal cognitive assessment (MOCA ≥ 21**).**

|  | | **Unstandard. coefficient** | **Standard error** | **Standard. coefficient β** | **t** | ***p*** | **Lower 95%CI** | **Upper 95%CI** | **Corrected R^2^** |
| --- | --- | --- | --- | --- | --- | --- | --- | --- | --- |
|  | constant | 64.947 | 25.508 |  | 2.546 | .014 | 13.806 | 116.088 | .524 |
|  | MOCA | -1.701 | .707 | -.228 | -2.405 | .020 | -3.119 | -.283 |  |
|  | NMS-Q | 1.247 | .389 | .330 | 3.203 | .002 | .467 | 2.028 |  |
|  | UPDRS-III | .310 | .066 | .486 | 4.681 | .000 | .177 | .443 |  |
|  | Age | -.439 | .217 | -.194 | -2.026 | .048 | -.874 | -.005 |  |

Entered independent variables: Beck Depression Inventory II (BD II), Montreal cognitive assessment (MOCA), non-motor symptoms questionnaire (NMS-Q), MDS-sponsored revision of the unified PD rating scale III (MDS-UPDRS-III), Hoehn & Yahr stage (HY), disease duration, sex, age, education level.

**Table S3.** MANOVA: Tukey-HSD post-hoc analysis on PDQ-39 domains in patients with normal Montreal cognitive assessment (MOCA ≥ 21).

| **PDQ-39 domain** (dependent variable) | **Factor**  ND (non-depressed),  STD (subthreshold depression)  D (depression) | | **Mean difference** | **Standard error** | ***p*** | **Lower 95%C** | **Upper 95%C** |
| --- | --- | --- | --- | --- | --- | --- | --- |
| Mobility | ND | STD | -16.394 | 13.466 | .445 | -48.339 | 15.551 |
|  |  | D | -33.710 | 14.220 | .050 | -67.443 | .024 |
|  | STD | ND | 16.394 | 13.466 | .445 | -15.551 | 48.339 |
|  |  | D | -17.315 | 14.955 | .481 | -52.793 | 18.162 |
| Activities of daily living | ND | STD | -29.025 | 13.160 | .074 | -60.242 | 2.192 |
|  |  | D | -43.223^*^ | 13.897 | .007 | -76.188 | -10.258 |
|  | STD | ND | 29.025 | 13.160 | .074 | -2.192 | 60.242 |
|  |  | D | -14.198 | 14.615 | .596 | -48.867 | 20.471 |
| Emotional well-being | ND | STD | -52.658^*^ | 11.773 | .000 | -80.586 | -24.730 |
|  |  | D | -88.393^*^ | 12.432 | .000 | -117.885 | -58.900 |
|  | STD | ND | 52.658^*^ | 11.773 | .000 | 24.729 | 80.586 |
|  |  | D | -35.735^*^ | 13.075 | .020 | -66.751 | -4.718 |
| Stigmatization | ND | STD | -18.371 | 13.920 | .387 | -51.392 | 14.651 |
|  |  | D | -36.140^*^ | 14.700 | .040 | -71.010 | -1.270 |
|  | STD | ND | 18.371 | 13.920 | .387 | -14.651 | 51.391 |
|  |  | D | -17.770 | 15.460 | .486 | -54.443 | 18.903 |
| Social Support | ND | STD | -14.726 | 12.144 | .448 | -43.533 | 14.081 |
|  |  | D | -34.361^*^ | 12.824 | .023 | -64.782 | -3.941 |
|  | STD | ND | 14.726 | 12.144 | .448 | -14.081 | 43.533 |
|  |  | D | -19.636 | 13.487 | .316 | -51.628 | 12.357 |
| Cognition | ND | STD | -38.317^*^ | 11.901 | .005 | -66.549 | -10.084 |
|  |  | D | -84.895^*^ | 12.568 | .000 | -114.708 | -55.082 |
|  | STD | ND | 38.317^*^ | 11.901 | .005 | 10.084 | 66.549 |
|  |  | D | -46.578^*^ | 13.218 | .002 | -77.933 | -15.224 |
| Communication | ND | STD | -9.869 | 13.540 | .747 | -41.989 | 22.252 |
|  |  | D | -36.925^*^ | 14.299 | .029 | -70.844 | -3.006 |
|  | STD | ND | 9.869 | 13.540 | .747 | -22.252 | 41.989 |
|  |  | D | -27.057 | 15.038 | .174 | -62.730 | 8.616 |
| Bodily Discomfort | ND | STD | -18.828 | 13.613 | .353 | -51.121 | 13.465 |
|  |  | D | -33.510 | 14.375 | .055 | -67.611 | .591 |
|  | STD | ND | 18.828 | 13.613 | .353 | -13.465 | 51.121 |
|  |  | D | -14.682 | 15.119 | .596 | -50.546 | 21.182 |
| *p<0.05 | | | | | | | |

**Table S4.** Clinical and sociodemographic characteristics of patients with low Montreal cognitive assessment (MOCA <21) based on depression level.

|  | | Nondepressed | | Subthreshold depression | | Depression | | ***p*** |
| --- | --- | --- | --- | --- | --- | --- | --- | --- |
|  | | ***n*** | **%** | ***n*** | **%** | ***n*** | **%** |  |
| Sex | female | 7 | 32.1 | 20 | 47.6 | 15 | 35.7 | 0.22 |
|  | male | 14 | 26.9 | 16 | 30.8 | 22 | 42.3 |  |
| Education level | low | 7 | 28.0 | 9 | 36.0 | 9 | 36.0 | 0.50 |
|  | middle | 4 | 19.0 | 6 | 28.6 | 11 | 52.4 |  |
|  | normal | 7 | 21.9 | 16 | 50.0 | 9 | 28.1 |  |
|  | | **M** | **SD** | **M** | **SD** | **M** | **SD** | ***p*** |
| Age (years) | | 72.7 | 7.7 | 74.6 | 8.6 | 72.7 | 7.7 | 0.063 |
| Disease duration (years) | | 9.1^a^ | 7.3 | 5.9^b^ | 4.6 | 9.3^c^ | 5.4 | 0.014 |
| Hoehn and Yahr stage | | 3 | 0 | 3 | 1 | 3 | 0 | 0.616 |
| MDS-UPDRS III | | 76.1 | 35.2 | 72.9 | 26.6 | 79.3 | 24.4 | 0.728 |
| NMS-Quest | | 9.9^a^ | 4.8 | 11.4^b^ | 4.7 | 13.9^c^ | 4.4 | 0.008 |
| BDI II | | 4.9^a^ | 2.5 | 12.1^b^ | 1.8 | 21.9^c^ | 5.7 | <0.001 |
| MOCA | | 17.4 | 3.1 | 17.0 | 3.0 | 17.3 | 2.9 | 0.712 |
| Note: Beck Depression Inventory II (BDI II), Montreal cognitive assessment (MOCA), non-motor symptoms questionnaire (NMS-Q), MDS-sponsored revision of the unified PD rating scale III (MDS-UPDRS-III). Data are presented as mean and SD and for Hoehn and Yahr as median and IQR. Data were analyzed using the Kruskal–Wallis test with Bonferroni-correction. Post-hoc tests for Disease Duration *ab* = p 0.222, *bc* = 0.012, *ac* = 0.999; NMS-Q *ab* p = 0.766, *bc* p = 0.009, *ac* p = 0.110; for BDI II *ab* p < 0.001, *bc* p < 0.001, *ac* p < 0.001. | | | | | | | | |

**Table S5.** Comparison between patients with normal and low Montreal cognitive assessment (MOCA) score.

|  |  | **MOCA <21** | | | | **MOCA ≥ 21** | | | |
| --- | --- | --- | --- | --- | --- | --- | --- | --- | --- |
|  |  | **n** | **%** |  | | **n** | **%** |  | |
| Sex | female | 42 | 44.7 |  |  | 49 | 36.0 |  |  |
|  | male | 52 | 55.3 |  |  | 87 | 64.0 |  |  |
| Education level | low | 25 | 32.1 |  |  | 17 | 13.6 |  |  |
|  | middle | 21 | 26.9 |  |  | 45 | 36.0 |  |  |
|  | normal | 32 | 41.0 |  |  | 63 | 50.4 |  |  |
| Hoehn & Yahr | 1 | 3 | 3.2 |  |  | 7 | 5.1 |  |  |
|  | 2 | 8 | 8.5 |  |  | 29 | 21.3 |  |  |
|  | 3 | 59 | 62.8 |  |  | 82 | 60.3 |  |  |
|  | 4 | 20 | 21.3 |  |  | 17 | 12.5 |  |  |
|  | 5 | 4 | 4.3 |  |  | 1 | 0.7 |  |  |
|  | | **M** | **SD** | **Lower**  **95%CI** | **Upper 95%CI** | **M** | **SD** | **Lower**  **95%CI** | **Upper 95%CI** |
| Age (years) | | 73.8 | 8.1 | 72.1 | 75.5 | 68.3 | 8.0 | 66.9 | 69.7 |
| Disease duration (years) | | 8.0 | 5.8 | 6.8 | 9.1 | 7.3 | 5.3 | 6.4 | 8.2 |
| MDS-UPDRS III | | 30.3 | 12.3 | 27.7 | 32.9 | 25.1 | 13.3 | 22.8 | 27.5 |
| NMS-Quest | | 12.1 | 4.8 | 11.1 | 13.1 | 9.9 | 5.0 | 9.0 | 10.8 |
| BDI II | | 14.3 | 7.7 | 12.7 | 15.9 | 12.1 | 8.7 | 10.6 | 13.6 |
| MOCA | | 17.2 | 3.0 | 16.6 | 17.8 | 24.4 | 2.5 | 23.9 | 24.8 |
| PDQ-39 | |  |  |  |  |  |  |  |  |
| Mobility | | 48.8 | 26.7 | 43.3 | 54.2 | 38.0 | 27.5 | 33.3 | 42.8 |
| Activities of daily living | | 41.5 | 25.2 | 36.3 | 46.6 | 31.4 | 25.3 | 27.1 | 35.7 |
| Emotional well-being | | 36.8 | 20.8 | 32.6 | 41.1 | 28.2 | 22.6 | 24.3 | 32.0 |
| Stigmatization | | 21.9 | 19.7 | 17.8 | 25.9 | 20.0 | 21.9 | 16.3 | 23.7 |
| Social Support | | 21.6 | 22.4 | 17.0 | 26.2 | 13.1 | 18.9 | 9.8 | 16.3 |
| Cognition | | 36.5 | 20.3 | 32.3 | 40.7 | 29.8 | 22.7 | 25.9 | 33.7 |
| Communication | | 30.6 | 21.8 | 26.2 | 35.1 | 25.3 | 23.7 | 21.2 | 29.4 |
| Bodily Discomfort | | 38.9 | 22.6 | 34.2 | 43.5 | 34.5 | 24.2 | 30.3 | 38.7 |
| Summary Index | | 34.6 | 15.8 | 31.4 | 37.8 | 27.4 | 16.9 | 24.5 | 30.4 |

Note: Beck Depression Inventory II (BDI II), Montreal cognitive assessment (MOCA), non-motor symptoms questionnaire (NMS-Q), MDS-sponsored revision of the unified PD rating scale III (MDS-UPDRS-III), Parkinson´s disease questionnaire-39 (PDQ-39).

**Table S6a.** Linear regression (backward selection): Association with PDQ-39 Summary Index in patients with low Montreal cognitive assessment (MOCA <21) score.

| **Model** | | **Unstand. coefficient** | **Standard error** | **Standard. coefficient β** | **t** | ***P*** | **Lower 95%CI** | **Upper 95%CI** | **Corrected R²** |
| --- | --- | --- | --- | --- | --- | --- | --- | --- | --- |
|  | Constant | -5.220 | 12.200 |  | -.428 | 670 | -29.579 | 19.138 | .315 |
|  | BDI II | .505 | .218 | .247 | 2.315 | .024 | .070 | .941 |  |
|  | MOCA | 1.208 | .557 | .228 | 2.169 | .034 | .096 | 2.320 |  |
|  | NMS-Q | 1.289 | .378 | .394 | 3.412 | .001 | .535 | 2.043 |  |
|  | UPDRS-III | .265 | .138 | .206 | 1.926 | .058 | -.010 | .540 |  |
|  | Sex | -7.638 | 3.250 | -.242 | -2.351 | .022 | -14.126 | -1.150 |  |

Entered independent variables: Beck Depression Inventory II (BDI II), Montreal cognitive assessment (MOCA), non-motor symptoms questionnaire (NMS-Q), MDS-sponsored revision of the unified PD rating scale III (MDS-UPDRS-III), Hoehn & Yahr stage (HY), disease duration, sex, age, education level.

**Table S6b.** Linear regression (backward selection): Association with PDQ-39 Summary Index excluding emotional well-being subdomain in patients with low Montreal cognitive assessment (MOCA <21) score**.**

| **Model** | | **Unstand. coefficient** | **Standard error** | **Standard. coefficient β** | **t** | ***P*** | **Lower 95%CI** | **Upper 95%CI** | **Corrected R²** |
| --- | --- | --- | --- | --- | --- | --- | --- | --- | --- |
|  | Constant | -10.625 | 10.072 |  | -1.055 | .299 | -31.095 | 9.844 | .435 |
|  | BDI II | .742 | .300 | .324 | 2.471 | .019 | .132 | 1.351 |  |
|  | NMS-Q | 1.192 | .464 | .379 | 2.571 | .015 | .250 | 2.134 |  |
|  | HY | 5.942 | 3.254 | .257 | 1.826 | .077 | -.671 | 12.555 |  |

Entered independent variables: Beck Depression Inventory II (BDI II), Montreal cognitive assessment (MOCA), non-motor symptoms questionnaire (NMS-Q), MDS-sponsored revision of the unified PD rating scale III (MDS-UPDRS-III), Hoehn & Yahr stage (HY), disease duration, sex, age, education level.

**Table S7.** Post-hoc univariate ANOVAs in patients with low Montreal cognitive assessment (MOCA <21) scores.

| **BDI II levels** | **F(2, 91)** | ***p*** | **partial η²** |
| --- | --- | --- | --- |
| Mobility | 4.530 | .013 | .091 |
| Activities of daily living | 3.473 | .035 | .071 |
| Emotional well-being | 12.993 | .000 | .222 |
| Stigmatization | .958 | .388 | .021 |
| Social Support | 10.017 | .000 | .180 |
| Cognition | 2.975 | .056 | .061 |
| Communication | 7.174 | .001 | .136 |
| Bodily Discomfort | 1.075 | .346 | .023 |

Note: Beck Depression Inventory II (BDI II).
